# The association between ethnicity and vaginal microbiota composition in Amsterdam, the Netherlands

**DOI:** 10.1371/journal.pone.0181135

**Published:** 2017-07-11

**Authors:** Hanneke Borgdorff, Charlotte van der Veer, Robin van Houdt, Catharina J. Alberts, Henry J. de Vries, Sylvia M. Bruisten, Marieke B. Snijder, Maria Prins, Suzanne E. Geerlings, Maarten F. Schim van der Loeff, Janneke H. H. M. van de Wijgert

**Affiliations:** 1 Amsterdam Institute for Global Health and Development, Amsterdam, The Netherlands; 2 Center for Infection and Immunity Amsterdam, Academic Medical Center, Amsterdam, The Netherlands; 3 Public Health Laboratory, Public Health Service of Amsterdam (GGD), Amsterdam, The Netherlands; 4 Department of Medical Microbiology and Infection Control, VU University Medical Center, Amsterdam, The Netherlands; 5 Department of Infectious Diseases, Public Health Service of Amsterdam (GGD), Amsterdam, the Netherlands; 6 Department of Dermatology, Academic Medical Center, Amsterdam, The Netherlands; 7 Department of Public Health, Academic Medical Center, Amsterdam, The Netherlands; 8 Department of Clinical Epidemiology, Biostatistics and Bioinformatics, Academic Medical Center, Amsterdam, The Netherlands; 9 Department of Internal Medicine, Division of Infectious Diseases, Academic Medical Center, Amsterdam, The Netherlands; 10 Department of Clinical Infection, Microbiology and Immunology, Institute of Infection and Global Health, University of Liverpool, Liverpool, United Kingdom; Fred Hutchinson Cancer Research Center, UNITED STATES

## Abstract

**Objective:**

To evaluate whether ethnicity is independently associated with vaginal microbiota (VMB) composition in women living in Amsterdam, the Netherlands, as has been shown for American women.

**Methods:**

Women (18–34 years, non-pregnant, N = 610) representing the six largest ethnic groups (Dutch, African Surinamese, South-Asian Surinamese, Turkish, Moroccan, and Ghanaian) were sampled from the population-based HELIUS study. Sampling was performed irrespective of health status or healthcare seeking behavior. DNA was extracted from self-sampled vaginal swabs and sequenced by Illumina MiSeq (16S rRNA gene V3-V4 region).

**Results:**

The overall prevalence of VMBs not dominated by lactobacilli was 38.5%: 32.2% had a VMB resembling bacterial vaginosis and another 6.2% had a VMB dominated by *Bifidobacteriaceae* (not including *Gardnerella vaginalis*), *Corynebacterium*, or pathobionts (streptococci, staphylococci, *Proteus* or *Enterobacteriaceae*). The most prevalent VMB in ethnically Dutch women was a *Lactobacillus crispatus*-dominated VMB, in African Surinamese and Ghanaian women a polybacterial *G*. *vaginalis*-containing VMB, and in the other ethnic groups a *L*. *iners*-dominated VMB. After adjustment for sociodemographic, behavioral and clinical factors, African Surinamese ethnicity (adjusted odds ratio (aOR) 5.1, 95% confidence interval (CI) 2.1–12.0) and Ghanaian ethnicity (aOR 4.8, 95% CI 1.8–12.6) were associated with having a polybacterial *G*. *vaginalis*-containing VMB, and African Surinamese ethnicity with a *L*. *iners*-dominated VMB (aOR 2.8, 95% CI 1.2–6.2). Shorter steady relationship duration, inconsistent condom use with casual partners, and not using hormonal contraception were also associated with having a polybacterial *G*. *vaginalis*-containing VMB, but human papillomavirus infection was not. Other sexually transmitted infections were uncommon.

**Conclusions:**

The overall prevalence of having a VMB not dominated by lactobacilli in this population-based cohort of women aged 18–34 years in Amsterdam was high (38.5%), and women of sub-Saharan African descent were significantly more likely to have a polybacterial *G*. *vaginalis*-containing VMB than Dutch women independent of modifiable behaviors.

## Introduction

The majority of women of reproductive age have a vaginal microbiota (VMB) dominated by lactobacilli [1]. VMB not dominated by lactobacilli are increasingly being recognized as a cause of adverse reproductive health outcomes, such as increased acquisition and transmission of HIV (reviewed in [2]) and preterm birth (reviewed in [3]). The clinical condition known as bacterial vaginosis (BV) is thought to be the most common type of VMB not dominated by lactobacilli [1, 4]. Molecular studies in the last decade have consistently identified the following anaerobes with high relative abundance in BV cases: *Gardnerella vaginalis*, *Atopobium vaginae*, BVAB1-3, *Mobiluncus* species, *Prevotella* species, *Sneathia/Leptotrichia* species, *Megasphaera* species, among others [3]. Other types of VMBs not dominated by lactobacilli, such as VMBs containing a high relative abundance of pathobionts, have also been identified but less commonly [1]. Until recently, many research groups lumped all VMBs not dominated by lactobacilli together into one community state type (CST IV), which was originally described by Ravel et al in 2011 [5]. An expert consultation organized by the U.S. National Institutes of Health (NIH) in 2015 called for more detailed descriptions of the various types of VMBs not dominated by lactobacilli and, eventually, subcategorization into multiple CSTs [6]. This is deemed necessary to improve our understanding of the etiology and pathogenesis of different CSTs and their sequelae, and to develop targeted interventions.

Another important finding from molecular studies in the last decade is that not all lactobacilli are equal [1]: a VMB dominated by *Lactobacillus crispatus* is considered more beneficial than a VMB dominated by *L*. *iners*, because the latter is associated with a higher risk of developing a VMB not dominated by lactobacilli [7] and a higher prevalence of sexually transmitted infections (STIs) [8].

Known risk factors for BV are unprotected sexual activity (reviewed in [9]), smoking [10], some types of vaginal cleansing practices (reviewed in [2]), the presence of STIs [11, 12], low levels of sex hormones [13–15], and African ethnicity (reviewed in [16]). Molecular studies in asymptomatic American women showed that African-American women are more likely to have a diverse VMB or a *L*. *iners*-dominated VMB than Caucasian or Asian-American women [5, 17–19]. Molecular studies have also shown that the prevalence of *L*. *crispatus*-dominated VMB is much lower in sub-Saharan Africa than in other parts of the world [20, 21]. However, whether the association between ethnicity and VMB composition is based on sociodemographic, behavioral, environmental, or genetic factors is still a matter of debate [22, 23]. Molecular studies conducted to date were small and did not adequately control for confounding factors, and few studies have been done in multi-ethnic European populations [1]. Comparisons within and between multi-ethnic populations in different parts of the world may provide the new insights required to disentangle the effects of sociodemographic, behavioral, environmental, and genetic factors on VMB composition.

In this study, we characterized VMB compositions of women representing the six largest ethnic groups in Amsterdam, the Netherlands. In keeping with the recommendation by the recent U.S. NIH consultation, we provide detailed descriptions of all diverse VMBs that we identified. We also determined factors associated with VMB composition with a focus on ethnicity.

## Materials and methods

### Study population

We used baseline data and samples from the HELIUS study, a multi-ethnic cohort study in Amsterdam, The Netherlands [24]. The HELIUS study aims to unravel the causes of the unequal burden of disease across ethnic groups residing in Amsterdam, and was approved by the Medical Ethics Committee of the Academic Medical Center in Amsterdam (protocol number: 10/100; amendment 10/100# 10.17.1729; NL32251.018.10). All participants provided written informed consent.

People aged 18–70 years were randomly sampled by ethnic origin (Dutch, Surinamese, Moroccan, Turkish, and Ghanaian) through the municipality register of Amsterdam [25]. For the Dutch sample, we invited people who were born in the Netherlands and whose parents were born in the Netherlands. For the Turkish sample, Turkish ethnicity was defined as: (a) born in Turkey and having at least one parent who was born in Turkey (first generation) or (b) born in the Netherlands but both parents were born in Turkey (second generation). A similar definition was used for the other ethnic minority groups (Surinamese, Moroccan and Ghanaian). After data collection, the Surinamese group was further stratified according to self-reported ethnic origin into ‘African’ (of West-African descent), ‘South-Asian’ (of North-Indian descent), ‘Javanese’, or ‘other’. In the VMB sub-study, we included African Surinamese women and women of North-Indian Surinamese or Javanese Surinamese descent (collectively referred to in this paper as South-Asian Surinamese) as two separate groups.

Between January 2011 and December 2015, HELIUS baseline data were collected for 24,789 participants. For the VMB sub-study, a subsample of women enrolled between 1 January 2011 and 31 December 2013 was selected: all women aged 18–34 years who had provided a self-sampled vaginal swab and had completed the questionnaire on sexual behavior were eligible for selection, and seven female participants per ethnic group per life year (or all available participants if fewer than seven were available) were randomly selected [26].

### Study procedures

Participants attended the study clinic for one study visit. They provided data on sociodemographics, behaviors, and medical history online or during the study visit. Participants who were unable to complete the questionnaire were offered assistance from a trained, ethnically matched interviewer. They were asked to bring a sample of their first morning urine to their study visit, which was tested by a urine reagent test strip (Combur 7, Roche). During the study visit, participants underwent a physical examination and biological sample collection. All women were asked to participate in the VMB sub-study by self-collecting a vaginal swab (Copan Diagnostics Inc., Murrieta, CA, USA) during the study visit, but refusal to do so did not render them ineligible for the HELIUS study. The vaginal swabs were kept at 2–8°C at the study site for up to six days, and then transported to, and stored at -20°C, at the Public Health Laboratory until testing. Women who were pregnant or less than 6 months postpartum were asked to postpone their HELIUS participation.

### VMB characterization

Each vaginal swab was eluted in 800 μl phosphate-buffered saline and divided into four aliquots. One aliquot each was tested for *Trichomonas vaginalis* (Aptima Trichomonas vaginalis assay, HOLOGIC, Bedford, MA, USA), *Chlamydia trachomatis* and *Neisseria gonorroeae* (Aptima Combo 2, HOLOGIC, Bedford, MA, USA), and human papillomavirus (HPV) (SPF10-PCR-DEIA/LiPA25 system version 1, DDL, Voorburg, the Netherlands) [26], and the fourth aliquot was used for VMB characterization. Samples were lysed using chemical lysis (lysozyme, mutanolysin (Sigma Aldrich, USA, St. Louis), and lysostaphin (AMBI, USA, New York)) and bead beating (MagnaPure, Roche Diagnostics, Switzerland, Basel), as described by Ravel [5]. Lysed samples were further treated using proteinase K and RNase A (Thermo Fisher, USA, Waltham). DNA was extracted using the ChemaGen extraction robot (PerkinElmer, Germany, Baesweiler). Dual indexed universal primers (319F and 806R) were used for PCR amplification of the V3-V4 regions of the 16S rRNA genes as described by Fadrosh [27]. PCR products were pooled and normalized, and purified with Agencourt AMPure XP magnetic beads (BeckmanCoulter, Fullerton, USA). Paired-end sequencing was performed on the Illumina MiSeq instrument (San Diego, CA, USA).

### Preprocessing of raw sequence data

Sequence data were preprocessed as described by Fadrosh [27] with minor modifications. Barcodes were trimmed off unaligned sequences. The unaligned sequences were then quality filtered by truncating reads with an average phred score <20 over a 30 bp sliding window with subsequent removal of sequences truncated to less than 75% of their original length as well as their paired sequences using Trimmomatic [28]. PANDAseq [29] was used for assembling paired sequences, error correction, and additional quality filtering: paired-end sequences that did not overlap or resulted in a sequence of <400 bp were removed. Sequences were matched to their corresponding barcode using the demultiplex tool in QIIME (version 1.8.0) [30] and removed when not matched to a barcode, being less than 75% of the original length after quality truncation, or having any ambiguous base calls. One mismatch was allowed during removal of PCR primer sequences (Tagcleaner [31]). The USEARCH tool in QIIME [32] was used for operational taxonomic unit (OTU) picking based on 97% sequence similarity and Greengenes version 13.8 was used as a reference for chimera detection [33]. Sequences were aligned to sequences in a vaginal reference package [34] using the PyNAST method in QIIME.

### Taxonomic classification

Taxonomic classification of OTUs was performed using two methods: 1) pplacer suite [35] with the vaginal reference package [34] as reference taxonomy and 2) RDP Classifier in QIIME with Greengenes version 13.8 [33] as reference taxonomy. The 16S rDNA BLAST function of the NCBI database was used when the two methods produced discordant results or when identification at species level was not possible (for OTUs with >1% relative abundance in at least one sample only). OTUs that were assigned to the same species were merged. Extremely rare OTUs (a total read count of less than 10) were removed. This resulted in 455 OTUs, with 96% mapping to species level, 3% to genus level, and 1% to higher taxonomic levels.

### Data analysis

Statistical analyses were performed using R version 3.1.3 (R Development Core Team, 2015) and STATA release 12 (StataCorp, College Station, TX, USA). All analyses were cross-sectional and contained one sample per participant. Samples with <100 high quality reads were excluded from subsequent analyses. Richness (number of OTUs) and diversity (Shannon Diversity Index) were calculated for each sample at a rarefied read count of 100. Hierarchical clustering was performed using Euclidean distance and complete linkage, and the resulting 20 clusters were visualized in a heatmap. Thirteen of these 20 clusters contained fewer than 10 samples. Clusters were therefore pooled based on microbiological characteristics to form meaningful VMB groups (see Table 1 for a detailed description of how this was done). The resulting eight VMB groups were described and their distribution across ethnic groups assessed. Sociodemographic, behavioral, and clinical characteristics were compared between HELIUS participants who did or did not provide a vaginal swab (to explore the magnitude and direction of participation bias), the six ethnic groups, and the three largest VMB groups (the other five VMB groups were too small to allow for multivariable modeling). The three largest VMB groups were: 1) *L*. *crispatus*-dominated VMB; 2) *L*. *iners*-dominated VMB; and 3) polybacterial *G*. *vaginalis*-containing VMB (Table 1). In unadjusted analyses comparing women who did or did not provide a vaginal swab, and the six ethnic groups, two-sided Chi-squared or Fishers’ exact tests were used for categorical data, and two-sided Kruskal Wallis tests for continuous data. Two separate multivariable logistic regression models were used to determine correlates of the *L*. *iners*-dominated VMB group and polybacterial *G*. *vaginalis*-containing VMB group, respectively, each compared to the *L*. *crispatus*-dominated VMB group. All covariates that were considered risk factors for a non-lactobacilli-dominated VMB a priori, and were associated with a VMB group in the unadjusted analyses at p<0.1, were included in the multivariable models. The proportion of women who used antibiotics in the past two weeks was small, and not statistically different among ethnic groups or VMB groups (see results). Furthermore, a sensitivity analysis excluding recent antibiotic users did not significantly alter our results and conclusions (data not shown).

**Table 1:** VMB cluster composition descriptions, Amsterdam, the Netherlands (2011–2015).

Clusters^1^	Description (*Percentages are relative abundances*)	N^2^	VMB group names^3^	N^2^
2	*L*. *crispatus*-dominated. 85 women had >90%, 44 had 50–90%, and 2 had <50% with *L*. *jensenii*.	131	***L*. *crispatus*-dominated**	**131**
1	*L*. *iners*-dominated. 94 women had >90%, 81 had 50–90%, and 12 had <50% with other lactobacilli.^4^	187	***L*. *iners*-dominated**	**187**
7	*L*. *gasseri*-dominated. 3 women had >90%, 6 had 50–90%, and 5 had <50% with other lactobacilli.	13	Other lactobacilli-dominated	18
9	*L*. *jensenii*-dominated. 2 women had >90%, and one had 50% *L*. *jensenii* and 45% *L*. *gasseri*.	3		
14	*L*. *delbrueckii*-dominated. Both women had 48–61% combined with 17–47% GV.	2		
4	Polybacterial with GV>AV or BVAB1. All women had GV (18–77%), 80 also had AV (1–30%), and 18 BVAB1 (3–18%). *Sneathia/Leptotrichia*, *Megasphaera* type 1, *Mobiluncus*, *Prevotella* (but always <20%), and *L*. *iners* were common^3^. Six women had some *L*. *gasseri*, two *L*. *jensenii*, and none *L*. *crispatus*.	98	**Polybacterial GV-containing VMB**	**134**
5	Polybacterial with AV>GV. All 31 women had GV (10–52%) and AV (26–69%), and 3 had <10% BVAB1. *Sneathia/Leptotrichia*, *Megasphaera* type 1, *Mobiluncus*, *Prevotella* (but always <20%), and *L*. *iners* were common.^5^ One woman each had some *L*. *jensenii* or *L*. *crispatus*.	31		
17	Polybacterial with BVAB1 and GV>AV. All 5 women had BVAB1 (40–47%), GV (4–16%), and AV (2–33%). *Sneathia/Leptotrichia*, *Megasphaera* type 1, *Mobiluncus*, *Prevotella* (but always <20%), and *L*. *iners* were common.^5^ No-one had *L*. *crispatus*.	5		
3	*G*. *vaginalis*-dominated. All 29 women had 75–100% GV.^6^	29	*G*. *vaginalis*-dominated	29
16	*Mobiluncus curtisii*-dominated (60%) with some GV, AV, and BVABs.	2	Other polybacterial anaerobic VMB	13
6A	Polybacterial with low abundance of GV, AV, and BVAB1: 7 women had >20% of *Prevotella* spp (21–34%), 3 had 37–44% *L*. *crispatus* with anaerobes, and one a mixture of anaerobes.	11		
11	*Bifidobacteriaceae*-dominated, not including GV (59–89%)	3	*Bifidobacteriaceae* or *Corynebacterium*-dominated	14
20	*Bifidobacterium*-dominated (75–99%)	3		
10	*Bifidobacterium breve*-dominated (40–89%)	4		
6B	Polybacterial with low abundance of GV, AV, and BVAB1, and >40% *Corynebacterium* spp (44–75%)	4		
18	*Streptococcus*–dominated (71%)	1	Pathobiont-dominated	20
8	*Streptococcus agalactiae*-dominated: 5 women had 62–97%, and 4 had 35–57% with other pathobionts, *Corynebacterium* spp, *Bifidobacteriaceae*, and/or lactobacilli.	9		
19	*Streptococcus anginosus*-dominated (69%)	1		
13	*Staphylococcus*-dominated (82%)	1		
12	*Escherichia coli*-dominated (83%)	1		
15	*Enterococcus faecalis*-dominated (55%), with some *S*. *agalactiae*, *S*. *anginosis*, and staphylococci.	2		
6C	Polybacterial with low abundance of GV, AV, and BVAB1, and >15% pathobionts (15–44%).	5		

AV = *Atopobium vaginae*, BVAB1 = BV-associated bacteria 1; GV = *Gardnerella vaginalis*, spp = species, VMB = vaginal microbiota.

^1^These numbers correspond to the 20 hierarchical clusters described in the results. In this table, clusters are organized into biologically meaningful VMB groups. Each hierarchical cluster was assigned to one biologically meaningful VMB group, except for cluster 6: this cluster was further subdivided into three groups (referred to as 6A-C), each of which was assigned to a different biologically meaningful VMB group. The hierarchical clustering clustered 6A-C together because of similar diversity combined with low abundance of GV, AV, and BVAB1, but they were otherwise biologically very different (see descriptions in the table).

^2^Number of samples containing the depicted VMB composition.

^3^VMB composition correlates were only determined for the three pooled clusters shown in bold.

^4^Two women also had >30% aerobes (37% *Psychrobacter*; 32% *Raoultella planticola*).

^5^Some women also had >1% *Actinomycetales*, *Anaerococcus* spp, other BVABs, *Clostridiaceae*, *Corynebacterium* spp (but <20%), *Dialister* spp, *Eggerthella*, *Finegoldia magna*, *Parvimonas micra*, *Peptoniphilus* spp, and *Veillionella* spp. >1% *Bifidobacterium* spp, *Mycoplasmas*, *Ureaplasmas*, and pathobionts were rare.

^6^Low levels of the same anaerobes as described for clusters 4, 5 and 17 were also often present.

## Results

### Study population

Of the 1,469 women aged 18–34 years who attended a HELIUS baseline visit before January 2014, 980 (67%) self-sampled a vaginal swab and 924 (63%) also completed the sexual behavior questionnaire. Up to seven women per ethnic group and per life year were randomly selected from these 924 women, which resulted in a sample size of 610 women. Dutch, African Surinamese, South-Asian Surinamese, and Ghanaian women were more likely to provide a vaginal swab (94%, 87%, 75%, and 71%, respectively) than Turkish and Moroccan women (37% and 49%, respectively; p<0.01; [26]).

The median age of the 610 selected women was 27 years (IQR 22–30), and did not differ between ethnic groups by design. However, other sociodemographic characteristics, substance use, reproductive health history, sexual behaviors, and clinical characteristics differed substantially (Table 2). Dutch women were more likely to have attained higher vocational or university education and to report use of tobacco, cannabis and alcohol, and less likely to report being religious and to have children. Turkish and Moroccan women were most likely to be married, Dutch and Surinamese women were most likely to currently use hormonal contraception, and Dutch and African Surinamese women were most likely to report sexual risk behaviors. Dutch and African Surinamese women also had the highest prevalence of any HPV and high risk HPV (both p<0.01) [24]. The prevalences of *Neisseria gonorrhoeae*, *Chlamydia trachomatis*, and *Trichomonas vaginalis* were low and not statistically significantly different across the ethnic groups.

**Table 2:** Characteristics of female participants by ethnic group, Amsterdam, the Netherlands (2011–2015).

	Dutch (n = 117)	South Asian Surinamese (n = 103)	African Surinamese (n = 116)	Ghanaian (n = 82)	Turkish (n = 89)	Moroccan (n = 103)	Total (n = 610)	*P*^1^
**Sociodemographic characteristics**								
Median age in years [IQR]	26 [22–30]	27 [23–31]	26 [22–30]	25 [21–30]	27 [23–31]	27 [23–31]	27 [22–30]	0.24
Currently married	5 (4.3)	17 (16.5)	3 (2.6)	6 (7.6)	40 (44.9)	36 (35.0)	107 (17.7)	<0.01
Higher vocational or university education	79 (67.5)	36 (35.0)	36 (31.3)	17 (21.0)	23 (25.8)	36 (35.0)	227 (37.3)	<0.01
Paid employment	86 (73.5)	58 (56.3)	68 (59.1)	40 (49.4)	59 (66.3)	58 (56.3)	369 (60.7)	<0.01
First generation migrant	NA	29 (28.2)	46 (39.7)	51 (62.2)	24 (27.0)	32 (31.1)	182 (29.8)	<0.01
Religious	9 (7.7)	77 (76.2)	80 (69.6)	73 (91.3)	74 (85.1)	99 (97.1)	412 (68.4)	<0.01
**Substance use**								
Self-reported current smoker	35 (29.9)	25 (24.5)	30 (26.1)	0 (0)	37 (41.6)	14 (13.6)	141 (23.2)	<0.01
Alcohol use in past 12 months	110 (94.0)	64 (62.7)	91 (78.4)	42 (51.2)	27 (30.3)	10 (9.7)	344 (56.5)	<0.01
Cannabis use in past 12 months	35 (29.9)	10 (9.8)	20 (17.2)	3 (3.7)	11 (12.4)	5 (4.9)	84 (13.8)	<0.01
**Reproductive health history**								
Median years since menarche [IQR]	13 [9–17]	15 [11–18]	14 [10–19]	12 [8–17]	14 [10–18]	14 [10–18]	14 [10–18]	0.02
≥1 child deliveries	6 (5.1)	28 (27.2)	28 (24.3)	29 (35.4)	37 (41.6)	37 (36.3)	165 (27.1)	<0.01
Current hormonal contraceptive use^2^	60 (51.3)	48 (46.6)	50 (43.1)	19 (23.8)	26 (29.2)	38 (37.3)	241 (39.7)	<0.01
**Sexual behavior**								
Currently having a steady partner	64 (54.7)	58 (56.3)	52 (44.8)	37 (45.1)	50 (56.8)	47 (46.1)	308 (50.7)	0.23
Median duration of relationship in months [IQR]	34.5 [13–60]	60 [24–92]	45 [13–86]	41 [26–100]	84 [38–120]	76 [50–120]	54 [25–96]	<0.01
Ever had sex, of which lifetime:	112 (95.7)	84 (81.6)	107 (92.2)	57 (69.5)	69 (77.5)	67 (65.0)	496 (81.3)	<0.01
Male sex partners 1	10 (8.9)	31 (36.9)	7 (6.7)	12 (21.1)	43 (63.2)	44 (66.7)	147 (29.9)	<0.01
2–5	42 (37.5)	39 (46.4)	49 (46.7)	32 (56.1)	14 (20.6)	15 (22.7)	191 (38.8)	
≥6	60 (53.6)	14 (16.7)	49 (46.7)	13 (22.8)	11 (16.2)	7 (10.6)	154 (31.3)	
Female sex partners ≥1	13 (11.6)	2 (2.4)	7 (6.6)	0 (0)	1 (1.4)	3 (4.5)	26 (5.3)	<0.01
Median age sexual debut in years [IQR]	16 [15–18]	18 [17–20]	16 [15–18]	18 [16–19]	20 [18–22]	19 [17–22]	18 [16–20]	<0.01
Sex in past six months:								
No recent sex	21 (18.8)	16 (19.0)	13 (12.3)	12 (21.1)	12 (17.4)	16 (23.9)	90 (18.2)	<0.01
Yes, only with steady partner	64 (57.1)	63 (75.0)	68 (64.2)	41 (71.9)	44 (63.8)	49 (73.1)	329 (66.5)	
Yes, casual partner +/- steady partner	27 (24.1)	5 (6.0)	25 (23.6)	4 (7.0)	13 (18.8)	2 (3.0)	76 (15.4)	
Condom use in past six months:								
No sex	21 (18.8)	16 (19.0)	13 (12.3)	12 (21.1)	12 (17.6)	16 (23.9)	90 (18.2)	0.56
Never / Mostly not / Sometimes	68 (60.7)	56 (66.7)	65 (61.3)	32 (56.1)	44 (64.7)	40 (59.7)	305 (61.7)	
Mostly / Always	23 (20.5)	12 (14.3)	28 (26.4)	13 (22.8)	12 (17.6)	11 (16.4)	99 (20.0)	
HIV-test in past 6 months	9 (7.7)	8 (7.8)	31 (26.7)	12 (14.8)	7 (7.9)	6 (5.8)	73 (12.0)	<0.01
Other STI-test in past 6 months	20 (17.1)	13 (12.6)	37 (31.9)	14 (17.3)	8 (9.0)	11 (10.7)	103 (16.9)	<0.01
Washing inside vagina ≥1x per week^3^	20 (17.1)	25 (24.3)	24 (20.7)	25 (30.9)	24 (27.0)	28 (27.2)	146 (24.0)	0.23
**Self-reported clinical characteristics**								
Antibiotic use in past 2 weeks	5 (4.3)	1 (1.0)	4 (3.4)	6 (7.4)	2 (2.3)	5 (4.9)	23 (3.8)	0.29
Urogenital symptoms in past month	47 (40.2)	43 (41.7)	52 (44.8)	27 (32.9)	36 (40.4)	49 (47.6)	254 (41.6)	0.45
≥3 vaginal infections per year	12 (10.3)	2 (1.9)	12 (10.3)	4 (4.9)	5 (5.6)	4 (3.9)	39 (6.4)	0.06
≥3 urinary tract infections per year	10 (8.5)	8 (7.8)	3 (2.6)	1 (1.2)	6 (6.7)	9 (8.7)	37 (6.1)	0.07
**Exam/test-based clinical characteristics**								
Diabetes^4^	0 (0)	3 (2.9)	0 (0)	1 (1.2)	1 (1.1)	1 (1.0)	6 (1.0)	0.22
Median body mass index in kg/m^2^ [IQR]	21.7 [20.1–23.3]	23.7 [20.8–28.1]	23.6 [21.6–26.9]	24.5 [22.2–27.9]	23.2 [21.6–26.2]	24.4 [22.5–27.8]	23.3 [21.3–26.5]	<0.01
Urinedipstick: Positive nitrite	4 (3.4)	5 (4.9)	6 (5.2)	1 (1.2)	8 (9.1)	6 (5.8)	30 (4.9)	0.28
Positive leukocytes^5^	14 (12.0)	23 (22.5)	23 (19.8)	13 (16.0)	19 (21.6)	31 (30.1)	123 (20.3)	0.03
Sexually transmitted infections, any	63 (53.8)	30 (29.1)	58 (50.0)	33 (40.2)	33 (37.1)	37 (35.9)	254 (41.6)	<0.01
Any HPV	61 (52.1)	29 (28.2)	56 (48.3)	31 (37.8)	32 (36.0)	34 (33.0)	243 (39.8)	<0.01
High risk HPV	48 (41.0)	18 (17.5)	36 (31.0)	22 (26.8)	26 (29.2)	27 (26.2)	177 (29.0)	<0.01
*Neisseria gonorrhoeae*	0 (0)	0 (0)	0 (0)	0 (0)	0 (0)	0 (0)	0 (0)	1.00
*Chlamydia trachomatis*	3 (2.6)	1 (1.0)	6 (5.2)	4 (4.9)	2 (2.2)	3 (2.9)	19 (3.1)	0.51
*Trichomonas vaginalis*	0 (0)	0 (0)	4 (3.4)	2 (2.4)	0 (0)	1 (1)	7 (1.1)	0.05

Cells contain number and column percentage, unless indicated otherwise. Abbreviations: IQR = interquartile range, NA = not applicable.

^1^ Kruskall Wallis for continuous variables and Chi^2^ or Fishers’ exact test for categorical variables.

^2^ Using hormonal pill (36%), vaginal ring (2%), implant (1%), hormonal patch (<1%), or injectable (<1%).

^3^ With water and soap (7%), vaginal washing products (17%), or other substances (<1%).

^4^ Self-reported diabetes, fasting glucose ≥ 7 mmol/l at physical exam, and/or reporting use of glucose-lowering medication.

^5^ +/++/+++ on urine dipstick. None of the variables had >5 missing values.

### VMB compositions in the overall study population

**Fig 1:**
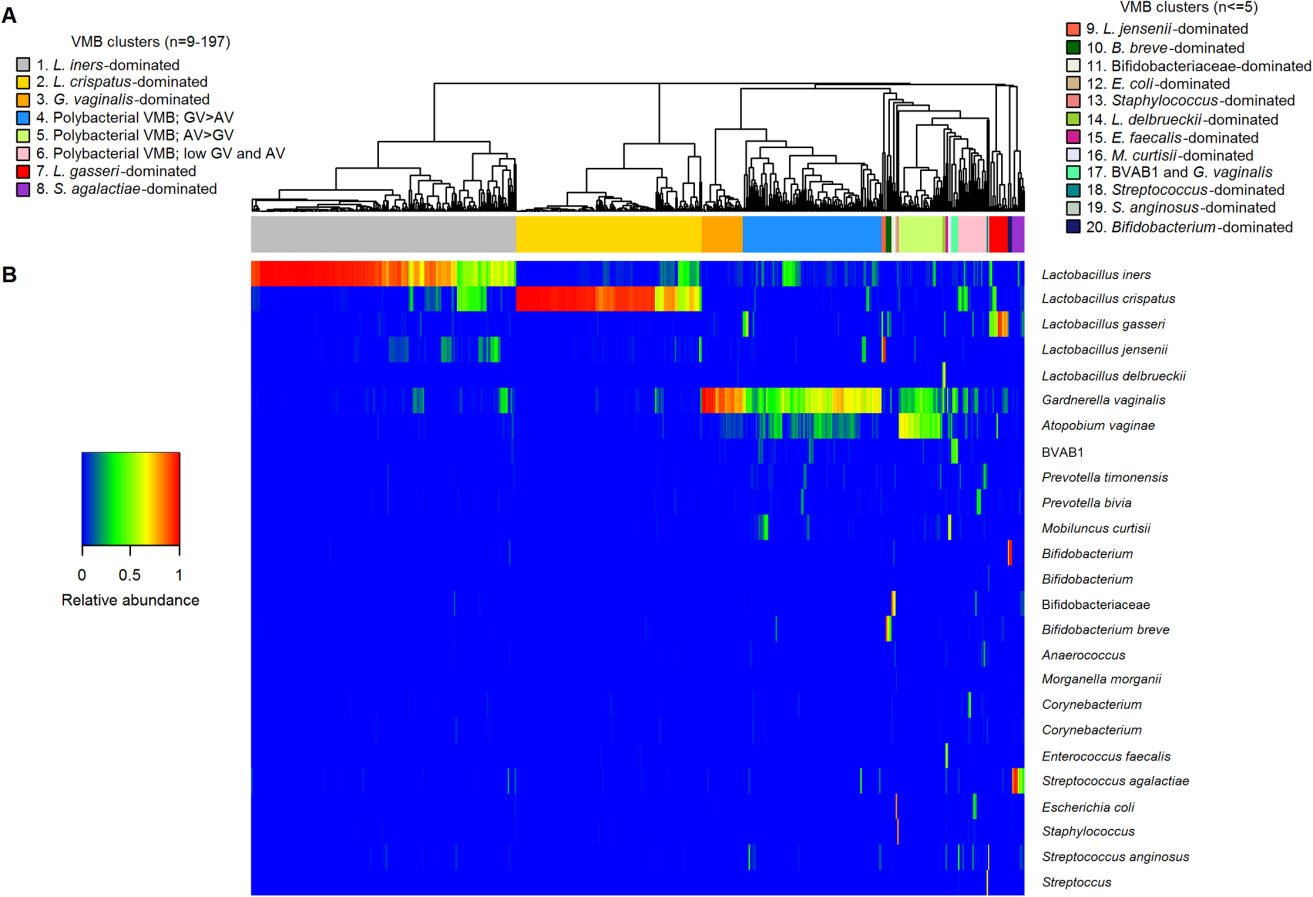
Hierarchical clustering of participants by VMB composition. AV = *Atopobium vaginae*; BVAB1 = BV-associated bacterium 1; GV = *Gardnerella vaginalis*; L = *Lactobacillus*; OTU = operational taxonomic unit; VMB = vaginal microbiota. A. The 20 VMB clusters obtained by hierarchical clustering of 546 vaginal samples. Clustering was based on the relative abundance of 455 OTUs. B. Heatmap showing the relative abundance of the most abundant OTUs using the color key on the left.

The median number of high quality sequences per sample was 25,392 (IQR 4,654–43,798). Samples with fewer than 100 sequences (n = 64) were discarded from further analyses; these were equally distributed across ethnic groups (data not shown). Hierarchical clustering of the remaining 546 samples resulted in 20 clusters (Fig 1, Table 1), which were pooled into eight microbiologically meaningful VMB groups as outlined in Table 1.

Three VMB groups were dominated by lactobacilli: *L*. *crispatus*-dominated VMB (n = 131, 24.0% of women), *L*. *iners*-dominated VMB (n = 187, 34.2%), and ‘other *Lactobacillus*-dominated’ VMB (pooling clusters dominated by *L*. *gasseri*, *L*. *jensenii*, and *L*. *delbrueckii*; n = 18, 3.3%). Most women in these groups had a VMB with more than 50% relative abundance of the relevant *Lactobacillus* species, and women with less than 50% of that species typically had additional *Lactobacillus* species. Other bacteria were sometimes present but in much smaller relative abundance, most commonly *G*. *vaginalis* and *Corynebacterium*. Pathobionts (streptoccoci, staphylococci, *Proteus*, and *Enterobacteriaceae*) were present in 14 women in a relative abundance of 1–14%. Overall, for all ethnic groups combined, 61.5% of the women had a lactobacilli-dominated VMB and 38.5% had another type of VMB.

Three of the five VMB groups that were not dominated by lactobacilli resembled BV. These included ‘polybacterial *G*. *vaginalis*-containing VMB*’* (pooling three polybacterial clusters with high relative abundance of *G*. *vaginalis*, *A*. *vaginae* and BVAB1 [4]; n = 134, 24.5% of women), ‘*G*. *vaginalis*-dominated VMB’ (n = 29, 5.3%), and ‘other anaerobic polybacterial VMB’ (pooling two polybacterial clusters with low relative abundance of *G*. *vaginalis*, *A*. *vaginae* and BVAB1 but high relative abundance of *Mobiluncus*, *Prevotella* species or other anaerobes; n = 13, 2.4%). All of these women had a polybacterial VMB characterized by the anaerobes that have typically been associated with BV (see Introduction and Table 1), often in combination with *L*. *iners* and several other minority taxa. *Bifidobacteriaceae* (not including *G*. *vaginalis*) were uncommon in these VMB groups. The final two of five VMB groups not dominated by lactobacilli included a VMB group dominated by *Bifidobacteriaceae* (not including *G*. *vaginalis*) or *Corynebacterium* (n = 14, 2.6%) and a VMB group dominated by pathobionts (n = 20, 3.7%). Overall, 32.2% of all women had a VMB resembling BV and another 6.2% had a VMB dominated by *Bifidobacteriaceae* (not including *G*. *vaginalis*), *Corynebacterium*, or pathobionts.

A total of 33 women (6.0%) distributed over all eight VMB groups had 1% or more relative abundance of a *Streptococcus* species, and an additional 18 women (3.3%) had 1% or more relative abundance of another pathobiont.

### Unadjusted associations between ethnicity and VMB groups

**Fig 2:**
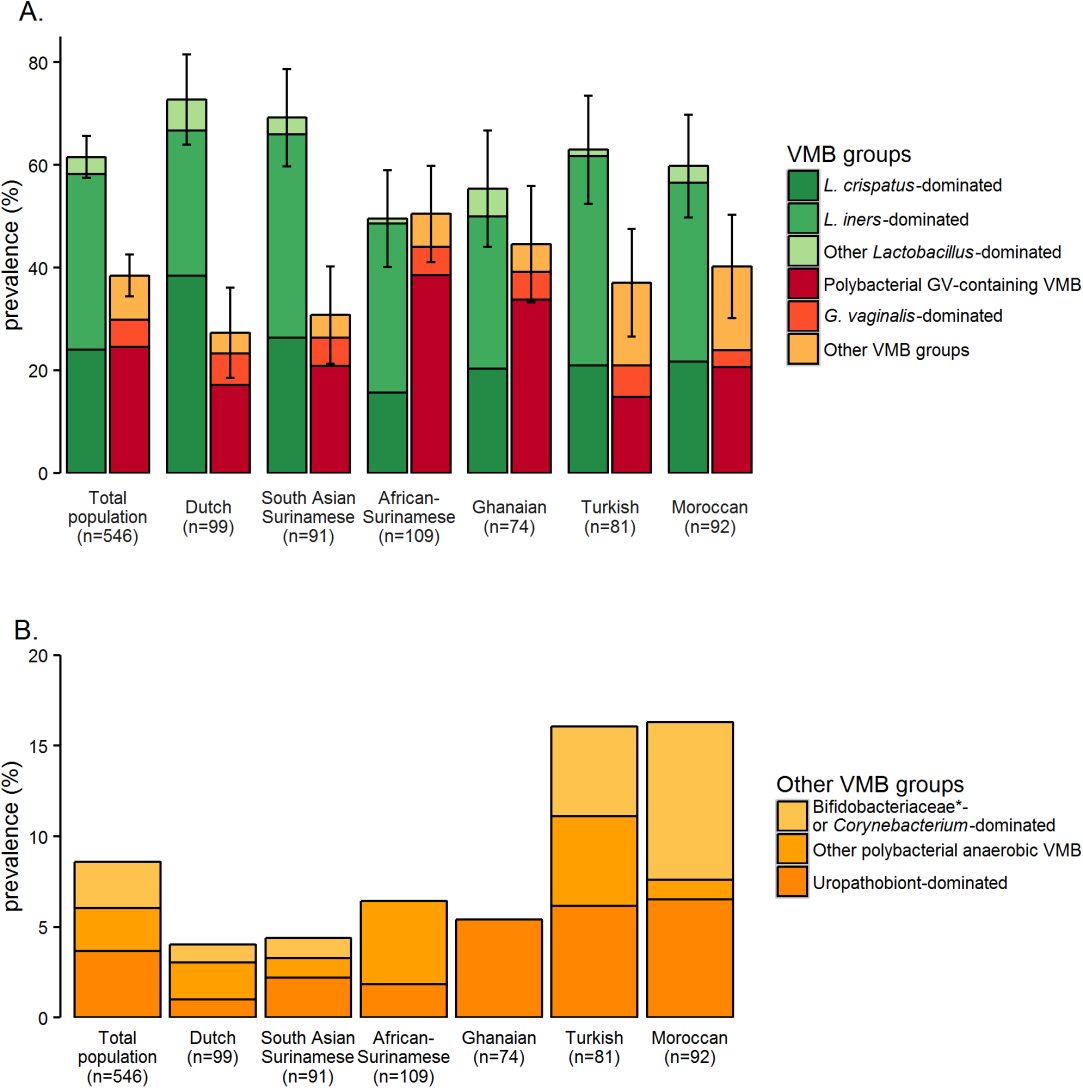
Prevalence of VMB groups by ethnic group. L = *Lactobacillus*; GV = *Gardnerella vaginalis*; VMB = vaginal microbiota. *Excluding GV. A. Prevalence of six VMB groups by ethnic group. The error bars show the 95% confidence intervals for the prevalence of the three *Lactobacillus*-dominated VMB groups combined and the three VMB groups not dominated by lactobacilli combined for each ethnic group. B. The ‘Other VMB’ group was further subdivided into three subgroups: The prevalence of these subgroups is shown by ethnic group.

The distribution of the eight VMB groups was significantly different among ethnic groups (Fig 2). The most prevalent VMB group in Dutch women was a *L*. *crispatus*-dominated VMB (38%), in South-Asian Surinamese, Turkish and Moroccan women a *L*. *iners*-dominated VMB (40%, 41%, and 35%, respectively), and in African Surinamese and Ghanaian women a polybacterial *G*. *vaginalis*-containing VMB (39% and 34%, respectively). Turkish and Moroccan women more often had a VMB dominated by *Bifidobacteriaceae* (not including *G*. *vaginalis*), *Corynebacterium* or pathobionts than women from the other ethnic groups (11% and 14%, respectively, versus 2–5%). When comparing the VMB group distribution of each ethnic group to the distribution in Dutch women (as shown in Fig 2), the Fisher’s exact p values were 0.41 for South Asian Surinamese women, <0.01 for African Surinamese women, 0.08 for Ghanaian women, <0.01 for Turkish women, and 0.01 for Moroccan women. The VMB of African Surinamese women had the highest richness (median 6.2, IQR 3.6–9.2) compared to a median richness of 4.2–5.2 in the other ethnic groups (overall Kruskal Wallis p = 0.02). The median Shannon Diversity Index [IQR] for Dutch women was 0.9 [0.3–1.5]. The median Shannon Diversity Indexes [IQR], and Kruskal Wallis p values for the other ethnic groups compared to Dutch women, were: South Asian Surinamese women 0.8 [0.2–1.4] (p = 0.31), African Surinamese women 1.5 [0.5–1.9] (p < 0.01), Ghanaian women 0.9 [0.2–1.7] (p = 0.83), Turkish women 1.0 [0.3–1.6] (p = 0.59), and Moroccan women 1.1 [0.4–1.7] (p = 0.16).

### VMB correlates in multivariable logistic regression models

VMB correlates were determined for the three largest of the eight VMB groups as described in the methods (Table 3). In adjusted analyses, being of African Surinamese and Ghanaian ethnicity, compared to Dutch ethnicity, was significantly associated with having a polybacterial *G*. *vaginalis*-containing VMB (adjusted odds ratio (aOR) 5.1, 95% confidence interval (CI) 2.1–12.0 and aOR 4.8, 95% CI 1.8–12.6, respectively), and being of African Surinamese ethnicity also with having a *L*. *iners*-dominated VMB (aOR 2.8, 95% CI 1.2–6.2). Other characteristics independently associated with having a polybacterial *G*. *vaginalis*-containing VMB at p<0.05 were being in a steady relationship for less than 2 years (aOR 3.5, 95% CI 1.4–8.5), inconsistent condom use with a casual sex partner in the past six months (aOR 3.2, 95% CI 1.0–9.9), and currently not using hormonal contraception (aOR 0.5, 95% CI 0.3–0.8, for current use).

**Table 3:** VMB correlates: Multivariable logistic regression models comparing each VMB cluster to the *L*. *crispatus*-dominated cluster.

		*L*. *crispatus*-dominated (n = 131)	*L*. *iners*-dominated (n = 187)		Polybacterial GV-containing VMB (n = 134)	
		N (%)^1^	N (%)^1^	aOR (95% CI)^2^^,^^3^	N (%)^1^	aOR (95% CI)^2^^,^^4^
Ethnicity:	Dutch	38 (29)	28 (15)	ref	17 (13)	ref
	African Surinamese	17 (13)	36 (19)	2.8 (1.2–6.2)*	42 (31)	5.1 (2.1–12.0)*
	S-Asian Surinamese	24 (18)	36 (19)	1.9 (0.9–4.0)	19 (14)	1.6 (0.7–4.0)
	Turkish	17 (13)	33 (18)	**2.1 (0.9–4.9)**	12 (9)	1.1 (0.4–3.0)
	Moroccan	20 (15)	32 (17)	**2.1 (0.9–5.0)**	19 (14)	**2.4 (0.9–6.3)**
	Ghanaian	15 (11)	22 (12)	2.1 (0.8–5.4)	25 (19)	4.8 (1.8–12.6)*
Currently married		14 (11)	39 (21)	0.9 (0.3–2.4)		
≥1 child births		23 (18)	60 (32)	1.5 (0.7–2.9)		
Current hormonal contraception use		64 (49)	72 (39)	0.7 (0.4–1.1)	42 (31)	0.5 (0.3–0.8)*
Alcohol use in past 12 months		87 (66)	102 (55)			
Steady relationship:	None	81 (62)	86 (46)	ref	63 (48)	ref
	<2 years	12 (9)	17 (9)	1.5 (0.6–3.7)	24 (18)	3.5 (1.4–8.5)*
	2–5 years	23 (18)	35 (19)	1.3 (0.6–2.8)	28 (21)	**2.2 (1.0–4.9)**
	≥6 years	14 (11)	47 (25)	**2.5 (0.9–6.8)**	17 (13)	1.9 (0.7–5.0)
Condom use in past six months:						
No recent sex		51 (39)	50 (27)	ref	38 (29)	ref
Consistent^5^ (steady/casual partner)		27 (21)	28 (15)	1.1 (0.5–2.2)	22 (17)	0.9 (0.4–2.1)
Inconsistent^6^ (steady partner only)		46 (35)	95 (51)	1.7 (0.9–3.2)	58 (44)	1.4 (0.7–3.0)
Inconsistent^6^ (casual +/- steady partner)		7 (5)	13 (7)	**2.5 (0.9–7.5)**	15 (11)	3.2 (1.0–9.9)*

aOR = adjusted odds ratio; CI = confidence interval; GV = *Gardnerella vaginalis*; ref = reference category; S-Asian = South Asian; VMB = vaginal microbiota.

Two separate multivariable logistic regression models were used to determine correlates of the *L*. *iners*-dominated VMB group and polybacterial *G*. *vaginalis*-containing VMB group, respectively, each compared to the *L*. *crispatus*-dominated VMB group.

^1^ N (%) is shown for all variables in the reference column, but in the other two columns, only for variables associated with the outcome (the *L*. *iners*-dominated VMB group or polybacterial *G*. *vaginalis*-containing VMB) at p<0.1 in unadjusted analyses (Fishers’ exact for categorical and Kruskal Wallis for continuous data). The models were not adjusted for any other variables.

^2^ aORs with 95% CI that were statistically significant at p<0.1 are shown in bold, and those significant at p<0.05 are also indicated with *.

^3,4^ Participants dropped from the multivariable model due to missing values: n = 8 (^3^) and n = 5 (^4^), respectively.

^5^ Reported as mostly/always.

^6^ Reported as never/mostly not/sometimes.

## Discussion

More than a third (38.5%) of the women in our population-based study in Amsterdam had a non-lactobacilli-dominated VMB, of which 83.6% resembled BV and 16.4% had a VMB dominated by *Bifidobacteriaceae* (not including *G*. *vaginalis*), *Corynebacterium*, or pathobionts. Women of sub-Saharan African descent (either African Surinamese or Ghanaian) were significantly more likely to have a polybacterial *G*. *vaginalis*-containing VMB, and African Surinamese women were significantly more likely to have a *L*. *iners*-dominated VMB, than Dutch women after controlling for sociodemographic factors, sexual risk behaviors, vaginal cleansing practices, and hormonal contraceptive use.

While studies in multi-ethnic asymptomatic American populations have shown similar differences between African-American women and women of other ethnicities before [1, 5, 17–19], our study is the first to show this in a multi-ethnic population living in Europe. We note some differences between our study and the American multi-ethnic studies: VMB dominated by lactobacilli other than *L*. *crispatus* and *L*. *iners* (most notably *L*. *gasseri* and *L*. *jensenii*) were less common, and the overall prevalence of a VMB resembling BV in White and Asian women was higher, in our study. Hypotheses to explain the association between ethnicity and VMB composition include differences in sociodemographic, behavioral, environmental, and genetic factors. Our results suggest that genetic differences might play a role. First, our sampling strategy balanced ethnicity and age, and we controlled our analyses for a large number of other sociodemographic factors and behaviors. Second, our study included two groups of women of sub-Saharan African descent: African Surinamese and Ghanaian women. The African Surinamese went from West-Africa to Suriname, a former Dutch colony in South America, in the nineteenth century, and many migrated from Suriname to the Netherlands in the 1970s and 1980s due to an unstable political situation in Suriname. Ghanaians migrated to the Netherlands (and also to other European countries such as the UK and Germany) between 1974 and 1983, and again in the early 1990s, for economic and political reasons. Despite different migration histories, both groups had higher adjusted odds of having a polybacterial *G*. *vaginalis*-containing VMB compared to Dutch women of a similar magnitude (aOR 5.1 and 4.8, respectively). Macro-environmental factors are therefore unlikely to play a role, but the role of cultural habits that women and their sexual partners take with them when they migrate, such as diet and sexual, vaginal, and penile hygiene practices, cannot be ruled out (vaginal hygiene practices were, however, not associated with VMB group in our study). A few studies have suggested that genetic differences in vaginal mucosal immune responses might favor colonization by *L*. *iners* over *L*. *crispatus* in women of African descent [36–38] but this has not yet been sufficiently studied. We speculate that vaginal mucosal immune responses may have evolved differently in sub-Saharan Africa compared to Europe or the Americas as a result of differences in exposures at mucosal surfaces to types and quantities of pathogens and pathobionts.

The clustering we performed differentiated VMB groups not dominated by lactobacilli by bacterial diversity, the relative abundances of the BV-associated taxa *G*. *vaginalis*, *A*. *vaginae*, BVAB1, *Mobiluncus* species, and *Prevotella* species, and the relative abundance of *Bifidobacteriaceae* (not including *G*. *vaginalis*), *Corynebacterium*, and pathobionts. Others have also reported on VMB groups differentiated by bacterial diversity and relative abundance of typical BV-associated bacteria [1, 5, 17–19], but different research groups are not yet using consistent VMB descriptions and categorizations making between-study comparisons difficult. VMBs dominated by *Bifidobacteriaceae* (not including *G*. *vaginalis*), *Corynebacterium*, and pathobionts have been infrequently reported thus far [1, 19, 39]. A Russian study isolated four *Bifidobacterium* species (*B*. *bifidum*, *B*. *breve*, *B*. *adolescentis*, and *B*. *longum*) in vaginal specimens of healthy women of reproductive age, and these were capable of inhibiting pathobionts in vitro [39]. While the clinical significance of the *Corynebacterium* VMB group is unclear, VMBs dominated by pathobionts are likely associated with adverse reproductive health outcomes; culture-based studies have consistently identified these pathobionts as causes of pregnancy complications and neonatal sepsis [3, 40]. Of note, only 6% of women in our study had at least 1% relative abundance of a *Streptococcus* species in their VMB, whereas a recent systematic review of culture-based studies concluded that the mean prevalence of rectovaginal carriage of *S*. *agalactiae* was 17.9% worldwide and 19.0% in Europe [41]. These figures cannot be compared directly due to differences in sampling (vaginal versus rectovaginal) and laboratory methods (16S sequencing versus culture). However, further research is needed to explain these differences and ensure that pathobionts are accurately identified in molecular studies. In addition, in our study, pathobionts occurred across VMB groups, including lactobacilli-dominated VMB groups. A better understanding of when pathobionts become clinically relevant (for example, at which relative or absolute abundance), and whether VMB groups or community state types adequately capture those situations, is urgently needed.

The five VMB groups not dominated by lactobacilli in our study might require different types of treatment or combinations of treatments. The three VMB groups resembling BV might respond to metronidazole treatment, but the efficacy might differ depending on the relative abundances of key taxa such as *G*. *vaginalis*, *A*. *vaginae* and BVAB1 and/or the presence of a biofilm (*G*. *vaginalis* is thought to initiate a vaginal biofilm by providing the scaffolding needed for other anaerobes to colonize) [42]. In contrast, VMBs with high relative abundance of pathobionts would require a different antibiotic treatment [40]. Clinicians should therefore be enabled to differentiate between these conditions.

Our study was cross-sectional. The vaginal microbiota often fluctuate over time, and the potential confounders that we included in our models (sexual risk behaviors, vaginal cleansing practices, and hormonal contraceptive use) are also time-dependent. Furthermore, vaginal microbiota are often influenced by the menstrual cycle [1, 18], and menstrual cycle data were not collected in the HELIUS study. Transient vaginal dysbiosis may not be clinically relevant, and data on the proportions of women who have persistent vaginal dysbiosis are needed. It should also be noted that the data on the potential confounders were self-reported, and may therefore suffer from social desirability bias.

The HELIUS study selected women at random from among those registered in Amsterdam in the age range and ethnic groups of interest, and we selected our subsample from the HELIUS sample. However, our subsample did suffer from participation bias and possibly reporting bias. The Turkish and Moroccan participants, in particular, were much less likely than the other groups to self-sample a vaginal swab and to complete a sexual behavior questionnaire [26]. They were also less likely to report sexual risk behaviors, which could be due to actual differences in behaviors or in reporting of those behaviors. Our results related to the Turkish and Moroccan groups should therefore be interpreted with caution. However, we believe that these biases did not influence our main finding that women of sub-Saharan African descent were much more likely to have a suboptimal VMB after controlling for potential confounding factors than Dutch women. In the three ethnic groups included in those comparisons (Dutch, African Surinamese and Ghanaian), vaginal sampling rates were 94%, 87% and 71%, respectively. None of the characteristics that differed between those who self-sampled and those who did not were associated with VMB composition in multivariable models, except that Ghanaian women who self-sampled were more likely to be in a steady relationship of longer duration and to have had at least one child than Ghanaian women who declined self-sampling [data not shown]. This suggests that Ghanaian women who participated may have a lower risk of having a VMB not dominated by lactobacilli than those who did not, which only strengthens our main finding.

We conclude that the overall prevalence of VMB not dominated by lactobacilli in women aged 18–34 years in Amsterdam was high, and that women of sub-Saharan African descent were more likely to have a polybacterial *G*. *vaginalis*-containing VMB than Dutch women independent of modifiable behaviors. Further research is required to determine if these women are also more likely to have persistent vaginal dysbiosis, and whether this contributes to the higher prevalence of adverse reproductive outcomes in these groups. Our findings support hypotheses implicating the contribution of genetic causes to these differences, such as those related to differences in vaginal mucosal immune response, and these should be further investigated.
